# Toxicity of Neurons Treated with Herbicides and Neuroprotection by Mitochondria-Targeted Antioxidant SS31

**DOI:** 10.3390/ijerph8010203

**Published:** 2011-01-19

**Authors:** Tejaswini P. Reddy, Maria Manczak, Marcus J. Calkins, Peizhong Mao, Arubala P. Reddy, Ulziibat Shirendeb, Byung Park, P. Hemachandra Reddy

**Affiliations:** 1 Neurogenetics Laboratory, Division of Neuroscience, Oregon National Primate Research Center, Oregon Health & Science University, 505 NW 185th Avenue, Beaverton, OR 97006, USA; E-Mails: tejaswinireddy99@gmail.com (T.P.R.); manczakm@ohsu.edu (M.M.); calkinsm@ohsu.edu (M.J.C.); maop@ohsu.edu (P.M.); reddya@ohsu.edu (A.P.R.); shirende@ohsu.edu (U.S.); 2 Division of Biostatistics, Department of Public Health and Preventive Medicine, Oregon Health & Science University, 3181 SW Sam Jackson Park Road, Portland, OR 97239, USA; E-Mail: parkb@ohsu.edu; 3 Department of Physiology and Pharmacology, Oregon Health & Science University, 3181 SW Sam Jackson Park Road, Portland, OR 97239, USA

**Keywords:** Mitochondria-targeted antioxidant, herbicides, Picloram, Triclopyr, Szeto-Schiller peptide 31, mouse neuroblastoma cells, mouse primary hippocampal neurons, electron transport chain, oxidative stress

## Abstract

The purpose of this study was to determine the neurotoxicity of two commonly used herbicides: picloram and triclopyr and the neuroprotective effects of the mitochondria-targeted antioxidant, SS31. Using mouse neuroblastoma (N2a) cells and primary neurons from C57BL/6 mice, we investigated the toxicity of these herbicides, and protective effects of SS1 peptide against picloram and triclopyr toxicity. We measured total RNA content, cell viability and mRNA expression of peroxiredoxins, neuroprotective genes, mitochondrial-encoded electron transport chain (ETC) genes in N2a cells treated with herbicides and SS31. Using primary neurons from C57BL/6 mice, neuronal survival was studied in neurons treated with herbicides, in neurons pretreated with SS31 plus treated with herbicides, neurons treated with SS31 alone, and untreated neurons. Significantly decreased total RNA content, and cell viability in N2a cells treated with picloram and triclopyr were found compared to untreated N2a cells. Decreased mRNA expression of neuroprotective genes, and ETC genes in cells treated with herbicides was found compared to untreated cells. Decreased mRNA expression of peroxiredoxins 1–6 in N2a cells treated with picloram was found, suggesting that picloram affects the antioxidant enzymes in N2a cells. Immunofluorescence analysis of primary neurons revealed that decreased neuronal branching and degenerating neurons in neurons treated with picloram and triclopyr. However, neurons pretreated with SS31 prevented degenerative process caused by herbicides. Based on these results, we propose that herbicides—picloram and triclopyr appear to damage neurons, and the SS31 peptide appears to protect neurons from herbicide toxicity.

## 1. Introduction

Artificially synthesized, contact and systemic herbicides are widely used in the agricultural sector and in landscape turf management to prevent the growth of unwanted plants. Contact herbicides, such as paraquat, are fast-acting and destroy plant tissue upon contact. Systemic herbicides, such as glyphosate, are slow-acting and kill plants through the vascular system. Although synthesized herbicides are designed to target unwanted plants, many end up affecting other living species, including other plants, insects, rodents, and humans [1]. The side effects of these chemicals on living species, particularly on humans, are not well understood.

Recent studies have reported that commonly used herbicides and pesticides adversely affect humans [2–12]. For example, humans exposed to the herbicide paraquat have shown symptoms similar to those of Parkinson’s disease [11]. Paraquat is artificially synthesized, similar to the analgesic drug 1-methyl-4-phenyl-1,2,3,6-tetrahydro pyridine (MPTP), which metabolizes into 1-methyl-4-phenylpyridinium (MPP^+^), a chemical implicated in the development of Parkinson’s disease [13]. Many other artificially synthesized herbicides have also shown carcinogenic, mutagenic, and teratogenic effects on mammals, including humans.

The current study focused on the systemic, slow-acting herbicides picloram and triclopyr, which are among the most commonly used herbicides in the world [14–22]. Picloram (4-amino-3,5,6,-trichloropicolinic acid) is widely used for broadleaf and woody plant control. It comes from the pyridine family of compounds and is used in such open areas as pastures, rangelands, and forests [23]. Picloram is the active ingredient in weed-control products Tordon and Grazon [17–20]. Historically, picloram was infamously known for being mixed with 2,4-D to create Agent White, a defioliant used in the Vietnam War for plants that survived the toxicity of Agent Orange (2,4-D and 2,4,5-T). Agent Orange was found to have carcinogenic, mutagenic, and teratogenic effects on large human populations exposed to it [24]. Although picloram is understood to be a carcinogen [25], little is known about mechanisms of its neurotoxic effects on humans.

Triclopyr (3,5,6-trichloro-2-pyridinyloxyacetic acid) is the active ingredient in Garlon herbicides. Similar to picloram, triclopyr is also used widely for broadleaf and woody plant control. Triclopyr is a foliar systemic herbicide from the pyridine group of herbicides. It is known to have severe toxic effects on humans [14–16,21,22] and to have moderate toxicity in rat models [26].

Very little is known about the toxic and adverse effects of herbicides such as picloram and triclopyr, to the brain cells of rodents and humans. In the present study, we studied toxicity of herbicides using mouse neuroblastoma cells and primary neurons from C57BL/6 mice. Using N2a cells, we measured: (1) the total RNA content, (2) mRNA expression of genes that encode antioxidant enzymes—peroxiredoxins 1–6, neuroprotective genes (PGC1α, FOXO1, and NMDA receptor), and mitochondrial-encoded ETC genes (NADH subunit 1-complex I, cytochrome B (Cyt. B)-complex III, cytochrome c oxidase 1-complex IV, and ATPase 6-complex V, and (3) cell viability—in N2a cells treated and untreated with the herbicides and in N2a cells pretreated with SS31 and then treated with herbicides and also N2a cells treated with SS31. Using primary neurons from C57BL6 mice, we studied neurite outgrowth and neuronal survival: (1) in primary neurons treated with herbicides, (2) in primary neurons pretreated with mitochondria-targeted antioxidant SS31 plus treated with herbicides, (3) primary neurons treated with SS31, and (4) untreated primary neurons.

## 2. Materials and Methods

### SS31 peptide

We purchased the SS31 peptide, a mitochondria-targeted antioxidant, from AnaSpec, CA. The SS31 tetra-peptide was originally synthesized by Dr. Hazel H. Szeto in collaboration with Dr. Peter W. Schiller. Drs. Szeto and Schiller designed and synthesized four different peptides (SS31, SS02, SS20, and SS19) with the amino acids Dmt, D-Arg, Phe, and Lys, and with the Dmt residue [27]. The peptide SS31 was chosen for our study because it targets mitochondria and penetrates into cells and the mitochondria several hundred times [27]. When the SS31 peptide penetrates into mitochondria, tyrosine (Tyr) and dimethyltyrosine (Dmt) analogs from the peptides scavenge and diminish free radicals, such as H_2_O_2_, OH, and ONOO [27–29]. SS31 also prevents lipid peroxidation [27–29]. At low molar concentrations (e.g., 1 nM), SS31 is reported to protect mammalian cells, including neurons, from mitochondrial and other toxic insults [30]. Therefore, we used a 1 nM final concentration of SS31 in this study.

### Reagents and chemicals

We purchased and prepared a phosphate buffer saline (Invitrogen, CA), trypsin EDTA (Invitrogen), N2a media (1:1 of DMEM and OPTI-MEM, 1x Penicillin/Streptomycin, 5% Fetal Bovine Serum), picloram (Sigma-Aldrich, CA), triclopyr and MTT (Sigma-Aldrich, CA), Trizol (Invitrogen), chloroform, isopropyl alcohol, DEPC (Sigma-Aldrich).

### Primary neuronal cultures

The C57BL/6 mice were housed at the Oregon National Primate Research Center of Oregon Health & Science University (OHSU). They were originally purchased from Taconic Farms and then bred in our animal facility. The OHSU Institutional Animal Care and Use Committee approved all procedures for animal care according to guidelines set forth by the National Institutes of Health.

Primary neuronal cultures from these mice were prepared using methods described by Manczak *et al.* [30]. Briefly, mice postnatal day 1 were decapitated, and the brains were removed and placed in a room-temperature Hibernate®-A medium (Brain Bits, Springfield IL) supplemented with B-27 (Invitrogen) and 0.5mM GlutaMAX™ (Invitrogen). The hippocampus was then dissected for culturing. Hippocampus pairs from individual mice were minced into pieces less than 1 mm^3^ and transferred to a solution of papain (2 mg/mL; Worthington Biochemical Corp, Lakewood, NJ) that was dissolved in Hibernate®-A without calcium, but supplemented with 0.5mM GlutaMAX™. The tissue was digested for 30 min in a shaking water bath at 30 °C. Digested tissue was then removed to 2 mL HABG and triturated 10 times with a fire-polished, siliconized (Sigmacote; Sigma, St. Louis MO), 9 in glass pipette. Samples were allowed to settle by gravity for about 1 min. Then the supernatant containing dissociated neurons was removed to a fresh tube. An additional 2 mL HABG was added to the pellet, and the process was repeated until 6 mL dissociated neurons were collected. Neurons were centrifuged at 200 g for 2 min and then washed with 2 mL HABG. The pellets were resuspended in 2 mL Neurobasal™ (Invitrogen) supplemented with B-27 and 0.5 mM GlutaMAX™ (growth medium). The neurons were counted, plated at 500 neurons/mm^2^ on poly-D-lysine-coated coverslips, and placed into a 37 °C incubator at 5% CO_2_. One hr after plating, the growth medium was completely replaced. After 3 days *in vitro* and every 2 days thereafter for 10 days, one-half of the growth medium was changed.

### Treatment with mitochondria-targeted antioxidant SS31 and herbicides

One group of postnatal day 1 primary (cortical and hippocampal) neurons from C57BL/6 mice remained untreated, and others groups were treated with SS31 (1 nM), picloram (5 mM) triclopyr (3mM) and were used for experiments. To determine the preventive effects herbicide toxicity, we pretreated with SS31 (1 nM) for 6 h, and then treated with picloram (5 mM) and triclopyr (3 mM) for 48 h, and studied neurodegeneration or neuronal survival using a neuronal marker—Beta III tubulin. After the treatments, cells on coverslips were washed with warm PBS and then fixed in freshly prepared 4% paraformaldehyde in PBS for 10–15 minutes. Cells were then washed with PBS and permeabilized with 0.1% Triton-X100 in PBS. All incubations were performed in a humidified chamber. The cells were blocked with 1% blocking solution (Invitrogen) for 1 h at room temperature. The primary antibody for beta-III tubulin was diluted in a blocking solution 1:2,000 and incubated at room temperature for 2 h. The cells were then washed 3 times for 5 min with PBS. Alexa Fluor® 568 goat anti-mouse (Invitrogen), a secondary antibody, was diluted 1:500 in a blocking solution, and was then applied to the cells for 1 h at room temperature. The cells on the coverslips were washed 2 times for 10 min with PBS and then mounted onto glass slides using the mounting medium Prolong Gold (Invitrogen). The slides were allowed to dry for at least 24 h, and then we took images of cells, using a Zeiss widefield microscope with a 100x oil immersion lens.

### Mouse neuroblastoma cells

We studied the effects of herbicides on N2a cells, purchased from American Type Culture Collection (ATCC, Manassas, VA).

**Figure 1:**
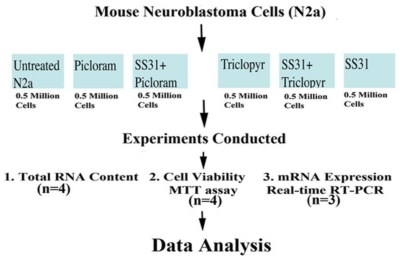
Experimental strategy of mouse neuroblastoma (N2a) cells pretreated with SS31 and then treated with the herbicides picloram and triclopyr.

Figure 1 illustrates the experimental strategy of our cell culture work, including treatments, number of cells per control (untreated N2a cells) and experimental groups (N2a cells treated with herbicides alone, and N2a cells pretreated with mitochondrial targeted antioxidant SS31 and then treated with herbicides), the number of replicates per experiment, and the number of experiments. As shown in Figure 1, half a million N2a cells were suspended per well into six-well plates. The cells were grown in a medium (1:1 DMEM and MEM, 5% FBS, 1x penicillin, and streptomycin) until 80% confluence in six-well plates. Then we treated them with a serum-free medium containing picloram (5 mM) and triclopyr (3 mM). In preliminary studies, the N2a cells started exhibiting signs of cell death, including losing projections, shrunken, and detaching from the surface of the flask, at a treatment dose of 5 mM for picloram and 3 mM for triclopyr. It is these concentrations that we used in our experiments.

To determine whether SS31 could protect the N2a cells from the toxicity of picloram and triclopyr, we pretreated them with SS31 (1 nM final concentration) in a serum-free medium) for 8 h, and then with 5 mM picloram and 3 mM triclopyr for 48 hrs. After this period, we harvested the N2a cells for RNA analysis (total RNA content and mRNA expression of mitochondrial ETC genes and neuroprotective genes) and MTT (3-(4,5-dimethylthiazol-2-yl)-2,5-diphenyl tetrazolium bromide) assay.

### RNA isolation

We isolated total RNA from the N2a cells: those cells that we had treated with picloram, those that were first pretreated with SS31 and then treated with picloram, those treated with triclopyr, those pretreated with SS31 and then treated with triclopyr, those treated with SS31 only, and those that were untreated. We used the standard TRIzol method to isolate RNA from all 8 samples. Briefly, tubes of N2a cells were defrosted and resuspended in 50 μL of PBS. One hundred μL of TRIzol was added to each tube, and the contents were mixed. One hundred μL of chloroform was then added to each tube, and the contents were mixed again. The tubes were centrifuged at 16,000 RPM, and the aqueous mixture was then transferred to newly labeled tubes. RNA was precipitated by the addition of 2 volumes of isopropyl alcohol. The tubes were centrifuged at 16,000 RPM, and the top layer of alcohol was discarded. The RNA pellet was washed with 70% alcohol. Ten μL DEPC-treated distilled water was added to each dried pellet. The optical density of each pellet was measured, to determine the RNA concentration.

### cDNA synthesis

As described by Manczak *et al.* [31], we synthesized cDNA using RNA from all N2a cell preparations. Using SYBR-Green chemistry-based quantitative RT-PCR, we measured mRNA expression of antioxidant enzyme proteins, including Prx1, Prx2, Prx3, Prx4, Prx5, Prx6, neuroprotective genes (PGC1α, FOX1, and NMDAR); and mitochondrially encoded genes (NADH sub1, COX1, Cyt. B, and ATP-6) for all preparations. Briefly, 1 μg DNAse-treated total RNA was used as starting material, to which we added the following: 1 μL oligo (dT), 1 μL 10 mM dNTPs, 4 μL 5× first-strand buffers, (4 μL 25 mM) MgCl_2_, (2 μL 0.1 M) DTT, and 1 μL RNAse out (Invitrogen). RNA, oligo (dT), and dNTPs were mixed first, heated at 65 °C for 5 min, and then chilled on ice until other components were added. The preparations were incubated at 42 °C for 2 min. Then 1 μL Superscript III (40 U/μL) (Invitrogen) was added to the preparations. They were incubated at 42 °C for 50 min. The reaction in each preparation was inactivated by heating the contents at 70 °C for 15 min.

### Quantitative RT-PCR analysis

Using primer express software (Applied Biosystems), we designed the oligonucleotide primers for the housekeeping genes β-actin and GAPDH; for mRNA expression of genes that encode antioxidant enzyme proteins, including Prx1, Prx2, Prx3, Prx4, Prx5 and Prx6; for neuroprotective genes (PGC1α, FOX1 and NMDAR); and for mitochondrially encoded genes (NADH sub1, COX1, Cyt. B, and ATP-6) for all preparations of the herbicides studied. The primer sequences and amplicon sizes are listed in Table 1.

Quantitative RT-PCR amplification reactions were carried out in an ABI Prism 7,900 sequence detection system (Applied Biosystems), in a 25-μL volume of total reaction mixture. The reaction mixture consisted of 1× PCR buffer containing SYBR-Green; 3 mM MgCl_2_; 100 nM of each primer; 200 nM each of dATP, dGTP, and dCTP; 400 nM dUTP; 0.01 U/μL AmpErase UNG; and 0.05 U/μL AmpliTaq Gold (ABI). Twenty nanograms of cDNA template were added to each reaction mixture.

To determine the unregulated endogenous reference gene in N2a cells treated with herbicides, we tested GAPDH. The C_T_-value—the cycle number at which the fluorescence generated within a reaction crosses the threshold within the linear phase of the amplification profile—is an important, quantitative parameter in RT-PCR analysis in the control and experimental samples because it determines the number of PCR cycles at which the PCR products start accumulating quantitatively in a real time [31]. All reactions were carried out in duplicate with a no template control. The PCR conditions were: 50 °C for 2 min, 95 °C for 10 min, followed by 40 cycles of 95 °C for 15 s, and 60 °C for 1 min. The fluorescent spectra were recorded during the elongation phase of each PCR cycle. To distinguish specific amplicons from non-specific amplifications, a dissociation curve was generated. The C_T_-values were calculated with the sequence-detection system (SDS) software V1.7 (Applied Biosystems) and with an automatic setting of base line, which was the average value of PCR from cycles 3–15, plus C_T_ generated 10 times its standard deviation. The amplification plots and C_T_-values were exported from the exponential phase of PCR directly into a Microsoft Excel worksheet for further analysis.

The mRNA transcript level was normalized against β-actin and GAPDH at each dilution. The standard curve was the normalized mRNA transcript level plotted against the log-value of the input cDNA concentration at each dilution. To quantify mRNA fold change, we used CT method of ABI [31]. Briefly, this method involved averaging duplicate samples taken as the C_T_-values for β-actin, GAPDH, and neuroprotective and mitochondrial encoded genes. We used GAPDH normalization in the present study because GAPDH CT values were similar for all groups of cells treated with the herbicides for all groups first pretreated with SS31 and then treated with the herbicides. The ΔC_T_-value was obtained by subtracting the average GAPDH C_T_-value from the average C_T_-value of neuroprotective and mitochondrial-encoded genes and peroxiredoxins. The present study used the average ΔC_T_ of untreated N2a cells as the calibrator. Fold change was calculated according to the formula 2^−(Δ ΔC_T_),^ where Δ ΔC_T_ was the difference between ΔC_T_ and the ΔC_T_ calibrator value [31].

### MTT Assay

For cell viability of N2a cells treated with picloram and triclopyr, and for N2a cells first pre-treated with SS31 and then the herbicides, we used an MTT assay, as described by Mosmann [32]. The purpose of the MTT assay was to determine the mitochondrial dehydrogenase cleavage activity of MTT in the N2a cells treated with herbicides. Two hr of MTT treatment was enough to determine mitochondrial respiration [33,34]. Briefly, after treatment with the herbicides or pre-treatment with SS31, 50 μL MTT assay solution (5 mg/mL) was added to each well. After 2 h, the solutions were removed, and 1 mL lysis buffer (20% SDS plus 50% DMSO) was added to each well. The plate was shaken for 5 min, and then the solutions were transferred to cuvettes. Blue Formosan, which was produced from MTT cleavage by active mitochondria dehydrogenase and dissolved in lysis buffer, was measured at a 570-nm wavelength. The optical density measurement of untreated N2a cells was assumed to be 100% for analysis of cell viability.

## 3. Statistical Considerations

Statistical analysis was performed using one-way analysis of variance (ANOVA) in order to determine the statistical significance between untreated (control) group, and experimental groups (N2a cells treated with herbicides alone, and N2a cells pretreated with mitochondria-targeted antioxidant, SS31 and then treated with herbicides) for the total RNA content, and cell viability. A permutation analysis was conducted in order to determine statistical significance between untreated (control) group, and experimental groups for total RNA content and cell viability.

## 4. Results

### Total RNA content

To determine whether picloram or triclopyr affected the total RNA content, we treated 0.5 million N2a cells with picloram (5 mM final concentration) and triclopyr (3 mM) for 48 h each and compared their total RNA content with that in equal numbers of untreated cells. We isolated total RNA content 4 times individually and calculated the mean total RNA for comparison. As shown in Table 2, we found significantly decreased total RNA content in the N2a cells treated with picloram (P < 0.0001) and triclopyr (P < 0.0001) compared to the untreated N2a cells (Table 2). However, in cells pretreated with SS31 and then treated with picloram, total RNA content was decreased (P < 0.0001) compared to untreated cells. In the N2a cells treated with only SS31, we found significantly increased total RNA content compared to the untreated N2a cells (Table 2). As shown in Table 2, 29.2 μg of total RNA was found in the untreated N2a cells, and total RNA increased to 32.1 μg in the N2a treated with SS31 (P < 0.002).

### Cell viability

To determine whether picloram and triclopyr are toxic to N2a cells and decrease cell viability, we used the MTT assay to analyze N2a cells treated with the herbicides and also N2a cells pretreated with SS31 and then treated with the herbicides. As shown in Table 3, cell viability was significantly decreased in the N2a cells treated with picloram (P < 0001), and triclopyr (P < 0.0001) compared to the untreated N2a cells. However, in the N2a cells pretreated with SS31 and then treated with the herbicides, cell viability was rescued by greater extent. In N2a cells pretreated with SS31 and no subsequent treatment, cell viability significantly increased (P < 0.0001), compared to untreated N2a cells.

### mRNA expression of mitochondrial-encoded genes in N2a cells treated with herbicides

As shown in Table 4, mRNA expression of mitochondrially encoded genes decreased for all genes in the N2a cells that were treated with picloram and triclopyr. However, in the N2a cells that were first pretreated with SS31 and then treated with picloram and triclopyr, mRNA expression levels were higher.

Among all mitochondrial-encoded genes that we studied, the complex I gene (NADH-subunit 1) decreased the most, followed by the complex V gene (ATPase6), then the complex IV gene (COX1), and lastly, the complex III gene (Cyt. B) in the N2a cells treated with herbicides. Among these herbicides, we found that mRNA expression of the mitochondrial encoded genes decreased the most in N2a cells treated with picloram, suggesting that picloram is more toxic to N2a cells. This decrease may have been the result of over-compensation by the increase in mRNA levels of the mitochondrial-encoded genes in N2a cells pretreated with SS31 and then further treated with herbicides. Overall, our findings of mRNA expression of mitochondria-encoded genes concurred with results of total RNA content and N2a cell viability.

### mRNA expression of antioxidant enzyme proteins (peroxiredoxins) in N2a cells treated with the herbicides

We measured mRNA expression of antioxidant enzyme proteins and peroxiredoxins 1–6 to determine the toxic effects of each of the two herbicides. We found decreased mRNA levels of Prx1 (52%), Prx3 (77%), Prx3 (35%), Prx4 (52%), Prx5 (89%), and Prx6 (7%) in the N2a cells treated with picloram compared to untreated N2a cells, indicating that picloram affects the mRNA expression of peroxiredoxins 1–6 (Table 4). However, in N2a cells pretreated with SS31 and then treated with picloram, we found that decreased mRNA levels of peroxiredoxins 1–6 increased to a greater extent (Table 4), suggesting that SS31 reduced the toxicity of picloram in N2a cells.

As shown in Table 4, in triclopyr-treated N2a cells, we found increased mRNA levels of Prx1 (by 12%), Prx2 (70%), Prx3 (30%), and Prx4 (41%), indicating that triclopyr enhanced peroxiredoxins activity in N2a cells. We found unchanged the mRNA expressions of Prx4 and Prx6 in N2a cells treated triclopyr compared to the mRNA expressions in untreated N2a cells. However, in N2a cells pretreated with SS31 and then treated with triclopyr, we found increased mRNA expressions of Prx1 Prx2, Prx3, Prx4, and Prx5 suggesting that SS31 boosted mitochondrial respiration in N2a cells.

### mRNA expression of neuroprotective genes in N2a cells treated with herbicides

We measured mRNA expression of the neuroprotective genes PGC1α, FOXO1, and NMDA in N2a cells treated with the herbicides only, and in N2a cells pretreated with SS31 and then treated with the herbicides.

As summarized in Table 4, in FOXO1, we found mRNA decreased by 68% in the picloram-treated cells and by 72% in the triclopyr-treated cells. In NMDAR, we found mRNA decreased by 50% in the picloram-treated cells and 79% in the triclopyr-treated cells. However, in N2a cells pretreated with SS31 and then treated with the herbicides, we found mRNA decreased in FOXO1 and NMDAR, suggesting that SS31 protected N2a cells from herbicide toxicity.

In N2a cells treated with picloram and triclopyr, mRNA expression in PGC1α was higher than the mRNA expression in the untreated N2a cells, indicating that herbicides did not influence the expression of mRNA in PGC1α. In N2a cells pre-treated with SS31, mRNA expression was higher suggesting that SS31 enhanced PGC1α levels and boosted overall mitochondrial function.

#### Herbicides and SS31 in primary neurons

**Figure 2:**
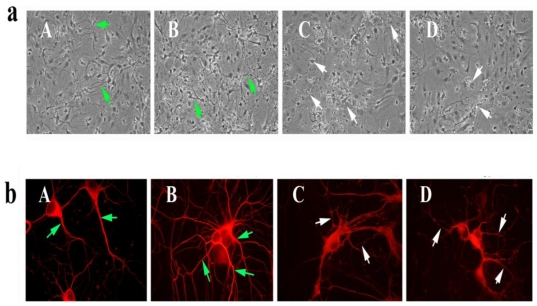
Primary neurons from C57BL/6 mice treated with picloram (C), triclopyr (D) and SS31 (B). The neurons treated with picloram (C) and triclopyr (D) exhibited damaged morphology: a loss of projections and network, compared to the untreated neurons (A). The neurons that were treated with SS31 (B) showed increased neuronal branching and network. Image **a** shows cortical neurons and photographs taken using phase-contrast microscope at 20x the original magnification. Image **b** shows immunofluorescence analysis of beta III tubulin antibody in hippocampal neurons treated with 1 nM SS31 (B), 5 mM picloram (C), and 3 mM triclopyr (D). Photographs were taken using fluorescence microscope at 100x the original magnification. Green arrows indicate intact/healthy neurons, and white arrows, degenerative neurons.

To determine the toxicity of picloram and triclopyr, we treated 7-day-old (or 7 DIV) primary neurons with picloram and triclopyr for 48 h and then studied the neuronal morphology using phase-contrast light microscopy. As shown in Figure 2, we found decreased neuronal branching and networks, and degenerated neurons in these primary neurons, compared to the neuronal morphology in the untreated primary neurons, indicating that picloram and triclopyr damage neuronal morphology. In contrast, the SS31-treated primary neurons showed increased neuronal branching and networks relative to the untreated primary neurons, suggesting that SS31 has the capacity to enhance neuronal viability.

**Figure 3:**
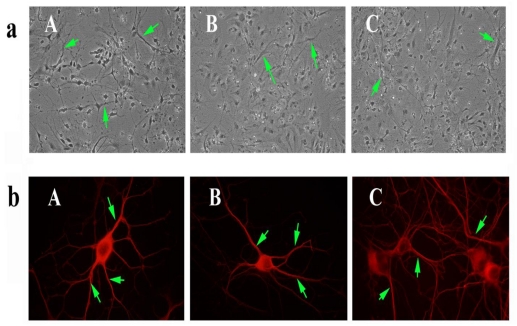
Primary neurons from mice pretreated with SS31 and then with picloram (B) and triclopyr (C). The neurons exhibited intact projections/network, similar to those in the untreated neurons (A). Image **a** shows primary cortical neurons and photographs taken with a phase-contrast microscope at 20x the original magnification. Image **b** shows immunofluorescence analysis of the beta III tubulin antibody in primary hippocampal neurons pretreated with 1 nM SS31 and then treated with 5 mM picloram (B) and 3 mM triclopyr (C). Pretreatment with SS31 prevented neuronal damage caused by picloram and triclopyr. Photographs were taken using a fluorescence microscope at 100x the original magnification. Green arrows indicate intact/healthy neurons, and white arrows, degenerative neurons.

To determine if SS31 protected cells against the toxicity of picloram and triclopyr on neuronal morphology, we pretreated primary neurons with SS31 and then treated them with picloram and triclopyr. As shown in Figure 3, those neurons pretreated with SS31 and then treated with triclopyr or picloram did not exhibit degeneration, indicating that SS31 prevents the toxicity of herbicides, picloram and triclopyr.

#### Immunofluorescence analysis using herbicides and SS31 in primary neurons

To determine neuronal morphology in primary hippocampal neurons exposed to picloram and triclopyr, we conducted immunofluorescence analysis using the cytoskeletal protein beta III tubulin antibody. We found decreased neuronal network and degenerating neurons in neurons treated with picloram and triclopyr. In contrast, in primary hippocampal neurons that we pretreated with SS31, and then treated with picloram and triclopyr, we found neuronal network and viability of neurons that resembled those of untreated neurons.

## 5. Discussion

We found significantly decreased total RNA content and decreased cell viability in N2a cells treated with picloram and triclopyr, compared total RNA content and cell viability in the untreated N2a cells. However, in the N2a cells that we first pretreated with SS31 and then treated with picloram and triclopyr, total RNA content and cell viability were basically not decreased, suggesting that SS31 had a neuroprotective effect against herbicide toxicity, thus preventing neuronal damage. Primary neurons that were treated with picloram and triclopyr showed losing network and signs of degeneration. However, primary neurons that were pretreated with SS31 and then treated with picloram and triclopyr showed increased neuronal networks. Thus, picloram and triclopyr damaged neuronal cells that were not when neurons were pretreated with SS31, indicating that SS31 protects neuronal cells against herbicides toxicity.

### Decreased cell viability in N2a cells treated with the herbicides

Mitochondrial respiration is a good measure to assess cell viability, particularly when cells under stress or exposed to toxins. Using MTT assay, we measured mitochondrial respiration in N2a cells treated with the herbicides, and found that the N2a cells treated with picloram and triclopyr showed decreases in mitochondrial respiration, indicating that the herbicides affect cell mitochondrial respiration (cell viability). However, in cells pretreated with SS31 and then treated with the herbicides, cell viability was increased greater extent—indicating that SS31 is capable of decreasing mitochondrial toxicity caused by herbicides. It is also interesting to observe that cells treated with only SS31 showed increased cell viability—suggesting that SS31 does not just prevent herbicide toxicity but also enhances mitochondrial respiration probably by decreasing free radicals and oxidative damage [35].

### Decreased mitochondrial-encoded gene expression in N2a cells treated with the herbicides

We measured the mRNA expression of mitochondrial-encoded genes in herbicide-treated and herbicide-untreated N2a cells to determine cell vitality since mitochondrial gene expressions are good indicators of energy in the cell mitochondrial metabolism [36]. Further, we also investigated the mRNA expression of mitochondrial-encoded genes to determine whether the mitochondria-targeted antioxidant SS31 increases mitochondrial activity in cells treated with SS31 and the herbicides. We found mRNA expression of mitochondrial-encoded genes decreased in N2a cells treated with the herbicides. Interestingly, we found complex 1 and complex V genes were down-regulated the most among all mitochondrial complexes studied, suggesting that complexes 1 and V are affected the most by the herbicides. In N2a cells pretreated with SS31, mitochondrial gene expressions were compensated for, to a large extent, suggesting that SS31 decreases mitochondrial free radicals and increases mitochondrial activity and function [29,35,37].

### Decreased mRNA expression of genes that encode antioxidant enzyme proteins in N2a cells treated with the herbicides

The purpose of measuring mRNA in N2a cells was to determine the activity of antioxidant enzyme proteins in cells treated with picloram and triclopyr. As described earlier, herbicides have mutagenic, carcinogenic, and teratogenic properties. However, their toxicity levels on neurons are less known. In the present study, we found decreased mRNA expression of peroxiredoxins in neurons treated with picloram. In triclopyr treated cells, we found unchanged or increased levels of the mRNA expression of peroxiredoxins. However, overall, neurons pretreated with SS31 and then treated with an herbicide showed increased levels of mRNA expression of antioxidant enzyme proteins.

Peroxiredoxins are a group of antioxidant enzymes present in all organisms. These antioxidant enzymes protect cells by regulating redox, phosphorylation, and oligomerization in cells [38]. There are 6 isoforms of peroxiredoxins (Prx1–6), and they neutralize H_2_O_2_ and participate in decreasing oxidative damage, which protects cells from various toxic insults. Peroxiredoxins are classified into three categories based on the position of the cystein and its number: (1) typical 2-Cys Prxs; (2) atypical 2-Cys Prxs; and (3) 1-Cys Prxs. These enzymes share the same basic catalytic mechanism, in which a redox-active cysteine in the active site is oxidized to a sulfenic acid by the peroxide substracter [38]. The relative abundance of Prx enzymes in mammalian cells appears to protect cellular components by reducing levels of peroxides in cells.

In the present study, we found decreased mRNA levels of peroxiredoxins 1–6 in the N2a cells treated with picloram, suggesting that picloram affects the mRNA expression of endogenous antioxidant enzymes, peroxiredoxins. In contrast, we found unchanged or slightly increased, the mRNA expression of peroxiredoxins in triclopyr-treated N2a cells, suggesting that used 3mM concentration of triclopyr does not affect peroxiredoxins expressions. It is possible that the concentrations that we used in treating N2a cells for triclopyr (3 mM) were too low to show an effect on mRNA expression, and at higher concentrations, they may decrease mRNA expression of peroxiredoxins. In the N2a cells pretreated with SS31 and then treated with the herbicides, we found increased mRNA levels for all peroxiredoxins, suggesting that SS31 decreases free radical production and mitochondrial oxidative damage, and enhances mitochondrial function and cell viability.

Very little is known about the role of peroxiredoxins in brain cells treated with herbicides. Our study is the first to describe the mRNA expression of peroxiredoxins in brain cells treated with picloram, and triclopyr. Further research is needed to understand the connection between increased mRNA expressions of peroxiredoxins in brain cells treated with picloram, in particular. In addition, further research is still needed to understand the mechanistic, biochemical link between herbicides and peroxiredoxins.

### Decreased expression of neuroprotective genes in N2a cells treated with the picloram and triclopyr

We measured mRNA in N2a cells treated with picloram and triclopyr and in untreated N2a cells to determine the neurotoxic effects of the herbicides. Although there are several neuroprotective genes in the genome, we selected PGC1α, NMDAR, and FOXO1 genes to study since they represent neuroprotection and determine the viability of the neuron. We found decreased mRNA expression of the 3 neuroprotective genes from the N2a cells that we had treated with the 2 herbicides. However, neurons treated with SS31, or pretreated SS31 and then treated with one of the herbicides showed increased expression of neuroprotective genes.

### PGC-1α

This transcription co-activator interacts with a range of transcription factors involved in a wide variety of biological responses, including adaptive thermogenesis and mitochondrial biogenesis of several tissues, such as brain tissues [39]. The overexpression of PGC1α was found to protect mammalian cells, including neurons, from mitochondrial oxidative damage by mediating gene expressions of transcription factors. Recently, lower-than-normal levels of PGC-1α expression were found in elderly persons and elderly persons with neurodegenerative diseases such as Alzheimer’s [40], and Huntington’s [41–43]. The connection between herbicide toxicity of neurons and PGC-1α expression is unknown. In the present study, we found PGC-1α expression is not affected by the low molar concentration of the herbicides. However, in N2a cells pretreated with SS31 and then treated with herbicides, PGC1α expression increased for each of the two herbicides, suggesting that SS31 boosts mitochondrial function and cell viability.

### FOXO1

FOXO1 is a fork head transcription factor of the FOXO subfamily, which plays an important role in cellular differentiation, proliferation, and metabolism [2,44–46]. A widely held theory is that chronic exposure of cells to elevated glucose concentrations causes a deterioration of β cell function. Glucose toxicity is thought to arise as a consequence of chronic oxidative stress, when intracellular glucose concentrations exceed the glycolytic capacity of the β cell. The over-expression of FOXO1 suppresses oxidative stress under hyperglycemia or abnormal glucose metabolism [47]. We found decreased mRNA expression of FOXO1 in the N2a cells that were treated with the herbicides, indicating that FOXO1 expression was affected by herbicides.

### N-methyl-D-aspartate receptor (NMDA receptor)

Recently, NMDAR activity in synapses was found to have neuroprotective effects by stimulating the antioxidant enzyme peroxiredoxin [48]. It is well-established that herbicides kill unwanted plants by inducing free radical production, mitochondrial damage, and cell death [17]. We found NMDAR expression was decreased in cells treated with herbicides. However, in N2a cells pretreated with SS31 and then treated with the herbicides, decreased NMDAR mRNA expression increased to some extent, suggesting that SS31 decreases free radical production and oxidative damage in cells It is possible that humans exposed to these herbicides may have adverse effects due to the effects of herbicides on free radicals and cell vitality.

### Decreased total RNA content in N2a cells treated with herbicides and altered expression of peroxiredoxins and electron transport chain genes

As described earlier, the total RNA content was decreased in cells treated with herbicides, picloram and triclopyr, and this decreased total RNA content may be responsible for decreased mRNA expression of antioxidant enzymes, peroxiredoxins 1–6, in cells treated with picloram and triclopyr. These findings clearly suggest that herbicides are toxic and influence mRNA expressions of peroxiredoxins. However, in cells pretreated with SS31 and then treated with herbicides, picloram and triclopyr the total RNA content was increased relative to picloram and triclopyr treated cells (Table 2), indicating that SS31 decrease cell toxicity and increase the total RNA content. This is truly reflected in our real-time PCR analysis of mRNA expression levels of peroxiredoxins 1–6, and electron transport chains genes (Table 4), suggesting that SS31 is protective against cell toxicity caused by herbicides, picloram and triclopyr.

### Primary neuronal cultures, herbicides and SS31

Our primary hippocampal neurons that we treated with picloram and triclopyr exhibited decreased neuronal branching and networks, and degenerating neurons. However, primary neurons treated with SS31 exhibited increased neuronal network and neuronal branching indicating SS31 has capability to decrease oxidative insults, and enhance neuronal viability. Further, in primary neurons, SS31 has increased neuronal network and branching, and in neurons pretreated with SS31, SS31 prevented neuronal damage caused by herbicides. These findings are in agreement with our previous observations that primary neurons from amyloid beta precursor protein transgenic mice were protected against amyloid beta toxicity when treated with SS31 [30]. In addition, SS31 increased RNA content, cell viability, and mRNA expression of endogenous antioxidant enzymes, peroxiredoxins in N2a cells. These results are consistent with SS31, which is known to decrease and to neutralize free radicals, and to protect neurons from oxidative insults. Overall, findings from our study suggest that SS31 is neuroprotective and may have therapeutic value against the toxicity of commonly used herbicides in an agriculture sector.

## Figures and Tables

**Table 1:** Oligonucleotide primers used for real-time RT-PCR analysis.

Gene	DNA Sequences (5′–3′)	PCR Product Size
Antioxidant Enzyme Proteins (Peroxiredoxins)		
Prx1	Forward Primer TGGCTCGACCCTGCTGATAG	61
	Reverse Primer GGAGCAGGATACCCAATTTTTG	
Prx2	Forward Primer CCCCTGAATATCCCTCTGCTT	57
	Reverse Primer CGCCGTAATTCTGGGACAA	
Prx3	Forward Primer GGCCCCATTTCTTGGAT	60
	Reverse Primer CAGGGCAGGCTAAGGGAAAG	
Prx4	Forward Primer CCTGTTGCGGACCGAATCT	55
	Reverse Primer GGGTCCGGAACCGTTCAT	
Prx5	Forward Primer CCCGATCAAGGTGGGAGAT	56
	Reverse Primer CCCGGTTCCCCTTCAAATA	
Prx6	Forward Primer TCTGGCAAAAAATACCTCCGTTA	58
	Reverse Primer GCCCCAATTTCCGCAAAG	
Neuroprotective Genes		
FOXOs1	Forward Primer CCCGTCCTAGGCACGAACT	69
	Reverse Primer ACGCGCCCAGAACTTAACTTC	
PGC1α	Forward Primer GGACAGTCTCCCCGTGGAT	57
	Reverse Primer TCCATCTGTCAGTGCATCAAATG	
NMDAR	Forward Primer GGTCAGTTCTGTCCTGCACATC	65
	Reverse Primer TGACTCTCCCGCGGAAAC	
Mitochondrial Electron Transport Chain Genes		
Complex I-NADHsubunit1	Forward Primer CGGGCCCCCTTCGAC	72
	Reverse Primer GGCCGGCTGCGTATTCT	
Complex II Cyt. B	Forward Primer TTATCGCGGCCCTAGCAA	70
	Reverse Primer TAATCCTGTTGGGTTGTTTGATCC	
Complex IV COX1	Forward Primer GAAGAGACAGTGTTTCATGTGGTGT	75
	Reverse Primer TCCTGGGCCTTTCAGGAATA	
Complex V ATPase-6	Forward Primer TGTGGAAGGAAGTGGGCAA	73
	Reverse Primer CCACTATGAGCTGGAGCCGT	
Housekeeping Genes		
Beta Actin	Forward Primer ACGGCCAGGTCATCACTATTC	65
	Reverse Primer AGGAAGGCTGGAAAAGAGCC	
GAPDH	Forward Primer TTCCCGTTCAGCTCTGGG	59
	Reverse Primer CCCTGCATCCACTGGTGC	

**Table 2:** The total RNA content in mouse neuroblastoma (N2a) cells pretreated with SS31 and then treated with picloram and triclopyr.

	Mean Total RNA (in μg)	SE	Difference	Dunnett p-value
Untreated N2a	29.3	0.45		
Picloram	12.7	0.45	−16.60	<0.0001
SS31 + picloram	23.6	0.45	−5.67	<0.0001
Triclopyr	9.2	0.45	−20.09	<0.0001
SS31 + triclopyr	20.0	0.45	−9.3	<0.0001
SS31	32.0	0.45	2.65	0.0023

Note: One-way ANOVA was used to compare neurons treated with the herbicides and those that were not treated. Exact p-values are provided. These p-values were obtained using permutation tests. Dunnett’s multiple comparison procedures with the untreated group as a control was used to adjust an experiment-wise error rate. Since we designed the study to compare each herbicide-treated group to the untreated group, we used Dunnett’s multiple comparison adjustment to compare each treatment to the control.

**Table 3:** Cell viability of mouse neuroblastoma cells pretreated with SS31 and then treated with picloram and triclopyr.

	Mean optical density of MTT assay solution	SE	Difference	Dunnett p-value
Untreated N2a	1.27	0.0266		
Picloram	0.66	0.0266	−0.61	<0.0001
SS31 + picloram	0.72	0.0266	−0.54	<0.0001
Triclopyr	0.66	0.0266	−0.60	<0.0001
SS31 + triclopyr	0.72	0.0266	−0.54	<0.0001
SS31	1.36	0.0266	0.09	0.1119

Note: Same note as in Table 2.

**Table 4:** Differences in mRNA fold changes in N2a cells pretreated with SS31 and then treated with picloram and triclopyr (SYBR-Green chemistry based on quantitative real-time RT-PCR).

Marker	N2a cells treated with picloram	N2a cells pretreated with SS31 and then treated with picloram	N2a cells treated with triclopyr	N2a cells pretreated with SS31 and then treated with triclopyr	N2a cells treated with SS31
**Antioxidants enzyme proteins (peroxiredoxins)**					
Prx1	0.48 (52% ⇓)	0.79 (21% ⇓)	1.12 (12% ⇑)	1.37 (37% ⇑)	1.20 (20% ⇑)
Prx2	0.23 (77% ⇓)	1.16 (16% ⇑)	1.7 (70% ⇑)	2.03 (103% ⇑)	1.10 (10% ⇑)
Prx3	0.35 (65% ⇓)	0.69 (31% ⇓)	1.3 (30% ⇑)	1.71 (71% ⇑)	1.00
Prx4	0.52 (52% ⇓)	0.90	0.99	2.01 (101% ⇑)	1.25 (25% ⇑)
Prx5	0.11 (89% ⇓)	0.37 (63% ⇓)	1.41 (41% ⇑)	1.48 (48% ⇑)	1.40 (40% ⇑)
Prx6	0.93	1.12 (12% ⇑)	1.02	0.93	1.30 (30%⇑)
**Neuroprotective genes**					
PGC1α	1.56 (56% ⇑)	1.55 (55% ⇑)	1.45 (45% ⇑)	1.89 (89% ⇑)	2.75 (175% ⇑)
FOXO1	0.32 (68% ⇓)	0.52 (48% ⇓)	0.28 (72% ⇓)	0.93 (7% ⇓)	0.45 (55% ⇓)
NMDAR	0.50 (50% ⇓)	0.65 (35% ⇓)	0.21 (79% ⇓)	0.93	0.89 (11% ⇓)
**Mitochondrial electron transport chain genes**					
NADH sub1	0.13 (87% ⇓)	0.22 (78% ⇓)	0.20 (80% ⇓)	0.36 (64% ⇓)	0.93
Cyt. B	0.54 (46% ⇓)	0.69 (31% ⇓)	0.74 (26% ⇓)	0.98 (2% ⇓)	1.94 (94% ⇑)
COX1	0.40 (60% ⇓)	0.75 (25% ⇓)	0.72 (28% ⇓)	1.14 (14% ⇑)	0.93
ATP-6	0.39 (61% ⇓)	0.58 (42% ⇓)	0.57 (43% ⇓)	0.89 (11% ⇓)	1.11 (11% ⇑)

Note: 1 fold mRNA expression means no change between experimental samples (N2a cells treated with picloram and triclopyr, N2a cells pretreated with SS31 and then treated with picloram and triclopyr, and N2a cells pretreated with only SS31) versus untreated N2a cells.

>1 fold mRNA expression indicates increased mRNA in untreated N2a cells.

<1 fold mRNA expression indicates decreased mRNA in untreated N2a cells.
